# Circulating 25-hydroxyvitamin D and lung cancer risk and survival: A dose–response meta-analysis of prospective cohort studies

**DOI:** 10.1097/MD.0000000000008613

**Published:** 2017-11-10

**Authors:** Qianqian Feng, Han Zhang, Zhengqin Dong, Yang Zhou, Jingping Ma

**Affiliations:** Department of Respiratory Medicine, The Second Clinical Medical College, Yangtze University, Jingzhou, Hubei, China.

**Keywords:** circulating 25-hydroxyvitamin D, dose–response relationship, lung cancer, meta-analysis

## Abstract

Lower serum level of 25-hydroxyvitamin D is associated with several negative outcomes. However, previous studies have indicated that 25-hydroxyvitamin D is associated with lung cancer risk and survival, but presented controversial results.

PubMed and Embase databases were searched update to August 2017 to identify and quantify the potential association between 25-hydroxyvitamin D and lung cancer risk and survival.

Seventeen eligible studies involving a total of 138,858 participants with 4368 incident cases were included in this meta-analysis. Our results showed statistically significant association between 25-hydroxyvitamin D and lung cancer risk and mortality. However, circulating 25-hydroxyvitamin D was not associated with overall lung cancer survival. Furthermore, compared with the lowest circulating 25-hydroxyvitamin D, the highest circulating 25-hydroxyvitamin D is significantly decreased risk of lung cancer risk in male and female. In addition, the highest circulating 25-hydroxyvitamin D was significantly associated with a lower risk in Caucasian and Asian. We also obtained the best fit at an inflection point of 10 nmol/L in piecewise regression analysis, increasing 10 nmol/L dose of circulating 25-hydroxyvitamin D was associated with an 8% reduction in the risk of lung cancer risk and an 7% reduction in the risk of lung cancer mortality. Subgroup meta-analyses in study quality, number of participants, and number of cases showed consistent with the primary findings.

The highest circulating 25-hydroxyvitamin D was associated with decreased lung cancer risk and mortality but not overall survival

## Abstract 2

Supplemental Digital Content is available in the text

## 1 Introduction

Lung cancer is one of the most serious and malignant diseases with the highest morbidity and mortality, and one of the most serious malignancies for population health and life.^[1]^ It accounts for the first incidence of all malignancies in male, with the second highest incidence in female, and the mortality rate account for the second of all malignancies.^[2]^ According to the American Cancer Association statistics, lung cancer mortality gradually increased, the overall cure rate for lung cancer has not improved significantly over the past decade.^[3]^ These data reveal the poor prognosis of lung cancer, and thus to prevent the occurrence of lung cancer is essential. Compared with many other cancers, there are a few identified risk factors for lung cancer, including asthma, chronic obstructive emphysema, pneumonia, tuberculosis, and atmospheric pollution.^[4,5]^ Meanwhile, the role of vitamin D has been recognized as an independently risk in several cancers, including lung cancer.^[6]^ Vitamin D mainly exists in the body in the form of circulating vitamin D, which is hydroxylated into 25-hydroxyvitamin D in the liver.^[7]^

Vitamin D is a fat-soluble vitamin, and the human body is mainly vitamin D_2_ and vitamin D_3_. The body's vitamin D is mainly derived from the body's own synthesis and animal food. The main function of vitamin D is to maintain the metabolism balance of human calcium and the formation of bone. In addition, vitamin D deficiency is closely related to abnormal immune function, cardiovascular disease, metabolic diseases, and tumors.^[8]^ Lower circulating 25-hydroxyvitamin D is a common condition in lung cancer. Also, lower vitamin D level is a potential reversible/modifiable risk factors for lung cancer.^[9]^

Previous studies have examined the relationship between circulating 25-hydroxyvitamin D and risk of cardiovascular disease, type 2 diabetes, and all-cause mortality, and have found higher circulating 25-hydroxyvitamin D is significantly reduce disease risk.^[8,10]^ Even though some studies supported higher circulating 25-hydroxyvitamin D significantly decrease lung cancer risk and survival. However, the result remains controversial. In addition, no study to quantitative assessed circulating 25-hydroxyvitamin D and lung cancer risk and survival. Thus, we performed this comprehensive dose–response meta-analysis to clarify and quantitative assessed the correlation between circulating 25-hydroxyvitamin D and lung cancer.

## 2 Methods

Our meta-analysis was conducted according to the Meta-analysis Of Observational Studies in Epidemiology checklist.^[11]^ There are no ethical issues involved in our study for our data were based on published studies.

### 2.1 Search strategy

Eligible studies were systematically searched of PubMed and Embase update to August 2017 examining the association between circulating 25-hydroxyvitamin D and lung cancer risk and survival, with keywords including “25-hydroxyvitamin D” [MeSH] OR “vitamin D” [MeSH] AND “lung cancer” [MeSH] OR “lung tumor” [MeSH]. We refer to the relevant original essays and commentary articles to determine further relevant research.

### 2.2 Study selection

Two independent researchers investigate information: outcome was lung cancer incidence and mortality. Moreover, we precluded nonhuman studies, reviews, editorials, and published letters. To ensure the correct identification of qualified research, the 2 researchers read the reports independently.

### 2.3 Data extraction

Use standardized data collection tables to extract data. Each eligible article information was extracted by 2 independent researchers. The following information was extracted: first author; publication year; age; country; sex; cases and participants; and relative risk or odds ratio. We collect the risk estimates with multivariable-adjusted. According to the Newcastle–Ottawa scale,^[12]^ quality assessment was performed for nonrandomized studies.

### 2.4 Statistical analysis

We pooled relative risk estimates to measure the association between circulating 25-hydroxyvitamin D and lung cancer risk and survival. Results in different subgroup of circulating 25-hydroxyvitamin D and lung cancer risk and survival were treated as 2 separate reports.

Due to different definitions cut-off points in the included studies for categories, using the method recommended by Greenland, Longnecker and Orsini et al^[13]^ by increase per 10 nmol/L circulating 25-hydroxyvitamin D. A flexible meta-regression based on restricted cubic spline function was used to fit the potential nonlinear trend, and generalized least-square method was used to estimate the parameters. This procedure treats circulating 25-hydroxyvitamin D (continuous data) as an independent variable and logRR of diseases as a dependent variable, with both tails of the curve restricted to linear.^[14]^

The between-study heterogeneity was assessed by Q-statistic (significance level at *P* ≤ .10) and the I^2^ statistic.^[15]^ STATA software 12.0 (STATA Corp, College Station, TX) was used in all analyses. *P* < .05 was considered significant for all tests.

## 3 Results

### 3.1 Literature search results

**Figure 1:**
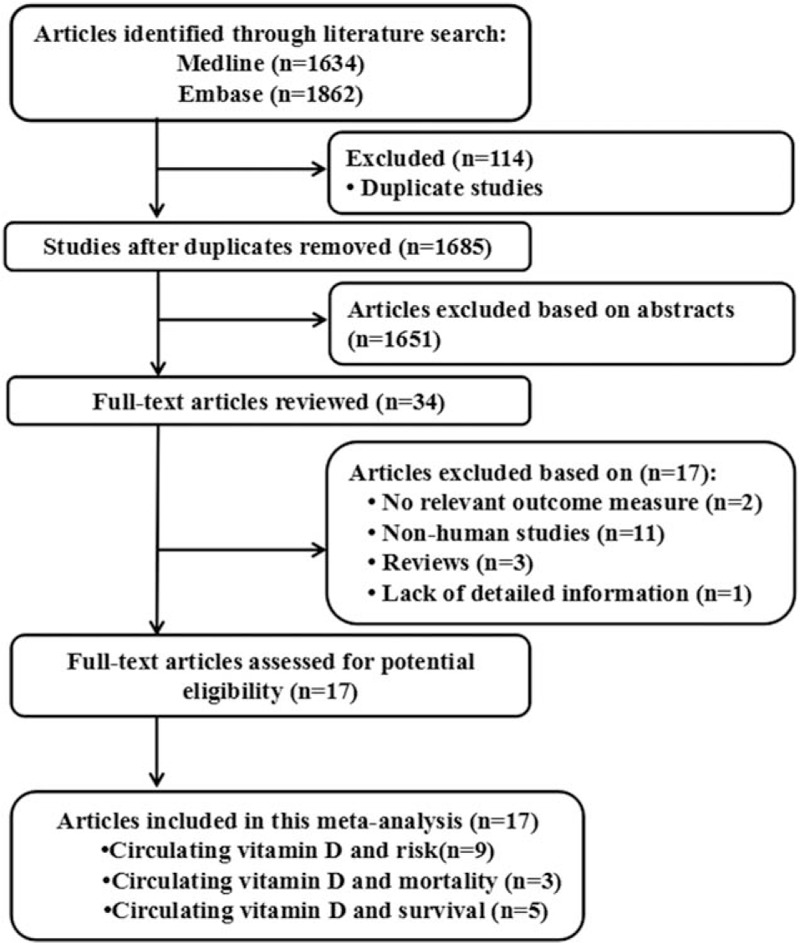
Flow diagram of the study selection process.

Figure 1 shows literature research and selection. A total of 1685 studies were retrieved (PubMed: 1634, Embase: 1862). After exclusion of studies, 17 studies were chosen, among the selected studies, 9 studies about the relationships between circulating 25-hydroxyvitamin D and lung cancer risk,^[16–24]^ 3 studies about the relationships between circulating 25-hydroxyvitamin D and lung cancer mortality,^[25–27]^ 5 studies about the relationships between circulating 25-hydroxyvitamin D and lung cancer survival,^[28–32]^ and the data were extracted. These studies were published update to August 2017.

### 3.2 Study characteristics

The characteristics of the included studies are shown in Tables 1 and 2. A total of 138,858 participants with 4368 incident cases were included in this meta-analysis.

**Table 1:**
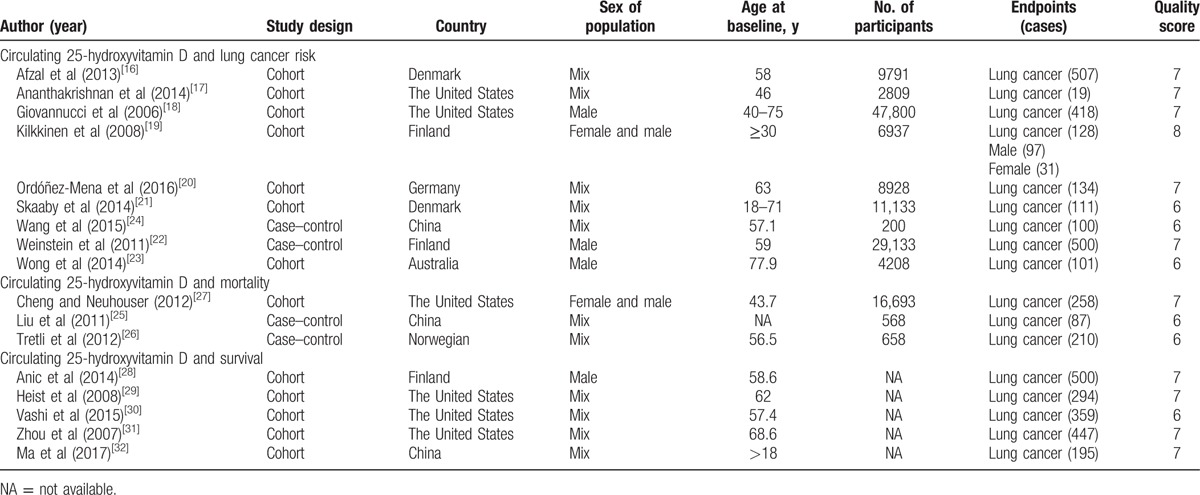
Characteristics of participants in included studies of circulating 25-hydroxyvitamin D in relation to lung cancer.

**Table 2:**
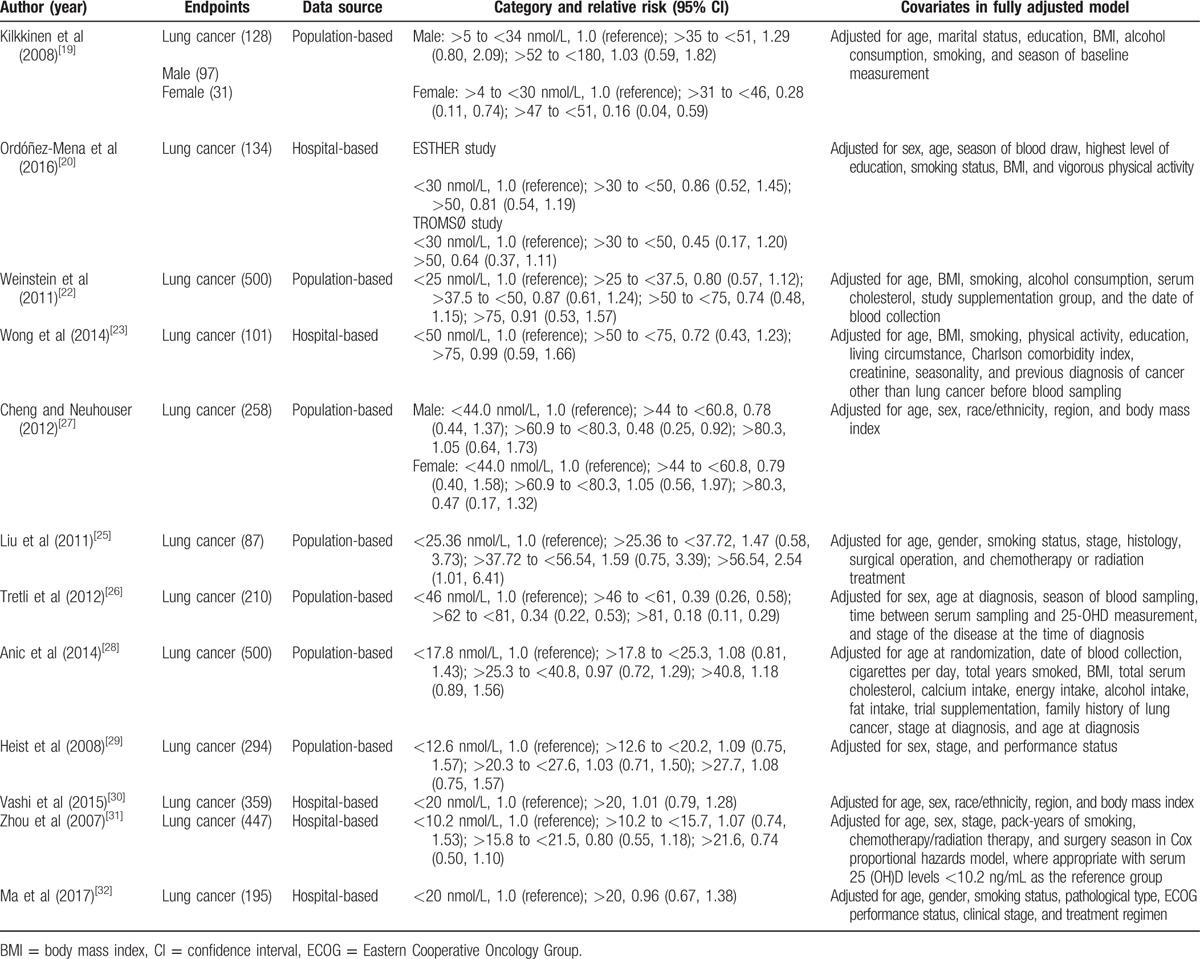
Outcomes and covariates of included studies of circulating 25-hydroxyvitamin D in relation to lung cancer.

### 3.3 Circulating 25-hydroxyvitamin D and lung cancer risk

**Figure 2:**
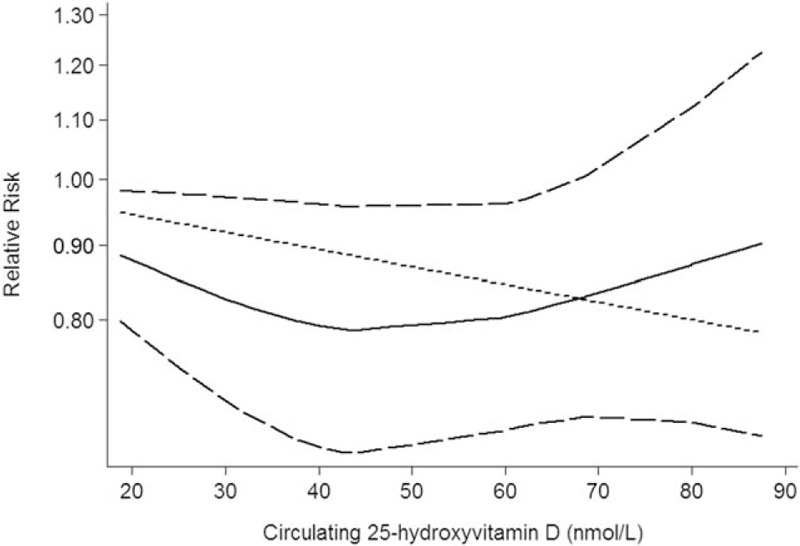
Circulating 25-hydroxyvitamin D is associated with lung cancer risk.

Nine studies including 11 independent reports investigated the association between circulating 25-hydroxyvitamin D and lung cancer risk. Higher circulating 25-hydroxyvitamin D was significantly decreased risk of lung cancer (relevant risk [RR]: 0.84; 95% confidence interval (CI): 0.74–0.95; *P* = .006; Table 3). Furthermore, higher circulating 25-hydroxyvitamin D was associated with a significantly decrement risk of lung cancer in female (odds ratio [OR] = 0.16, 95% CI: 0.04–0.59, *P* < .001; Table 3) and in male (OR = 0.82, 95% CI: 0.71–0.91, *P* < .001; Table 3). In addition, higher circulating 25-hydroxyvitamin D was significantly decreased risk in Caucasian (RR: 0.92; 95% CI: 0.88–0.95; *P* < .001; Table 3) and Asian (RR: 0.41; 95% CI: 0.19–0.91; *P* < .001; Table 3). We also obtained the best fit at an inflection point of 10 nmol/L in piecewise regression analysis, increasing 10 nmol/L of circulating 25-hydroxyvitamin D was associated with a 8% reduction in the risk of lung cancer, the summary relative risk of lung cancer risk for an per 10 nmol/L of circulating 25-hydroxyvitamin D was 0.92 (95% CI: 0.87–0.96, *P* < .001; Fig. 2).

**Table 3:**
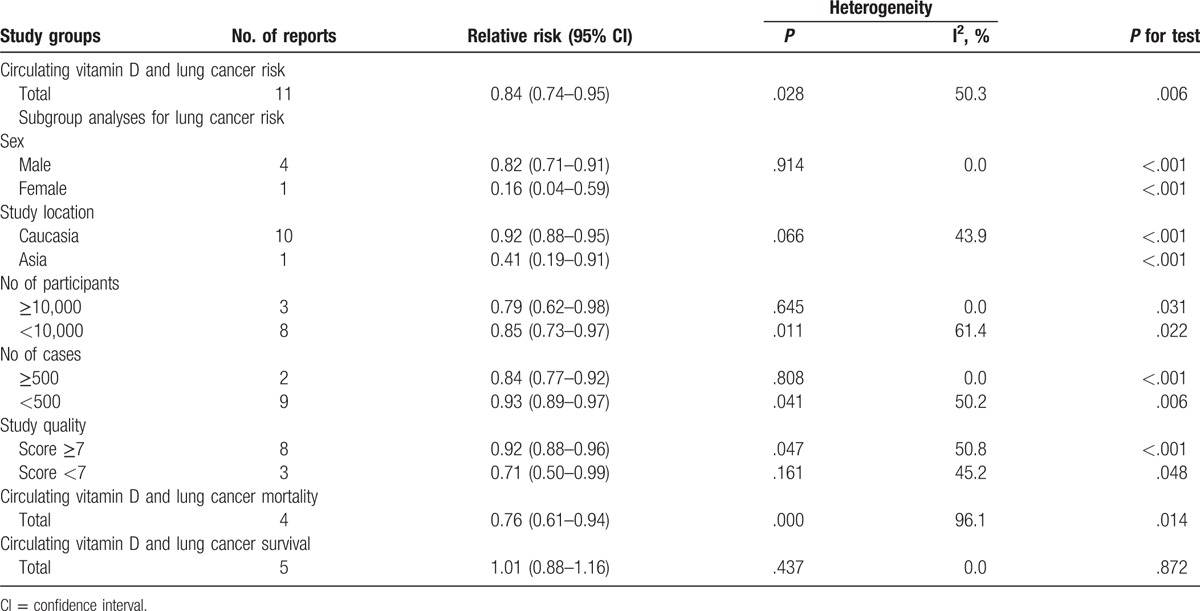
Stratified analyses of relative risk of lung cancer.

### 3.4 Circulating 25-hydroxyvitamin D and lung cancer mortality

**Figure 3:**
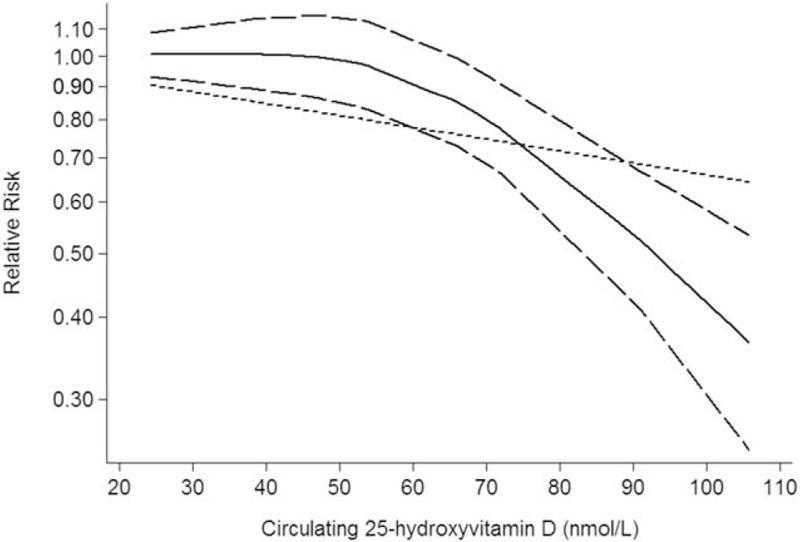
Circulating 25-hydroxyvitamin D is associated with lung cancer mortality.

Three studies including 4 independent reports investigated the association between circulating 25-hydroxyvitamin D and lung cancer mortality. Higher circulating 25-hydroxyvitamin D was significantly decreased risk of lung cancer mortality (RR: 0.76; 95% CI: 0.61–0.94; *P* = .014; Table 3). We also obtained the best fit at an inflection point of 10 nmol/L in piecewise regression analysis, increasing 10 nmol/L of circulating 25-hydroxyvitamin D was associated with a 7% reduction in lung cancer mortality, the summary relative risk of lung cancer mortality for an per 10 nmol/L of circulating 25-hydroxyvitamin D was 0.93 (95% CI: 0.88–0.96, *P* < .001; Fig. 3).

### 3.5 Circulating 25-hydroxyvitamin D and lung cancer survival

**Figure 4:**
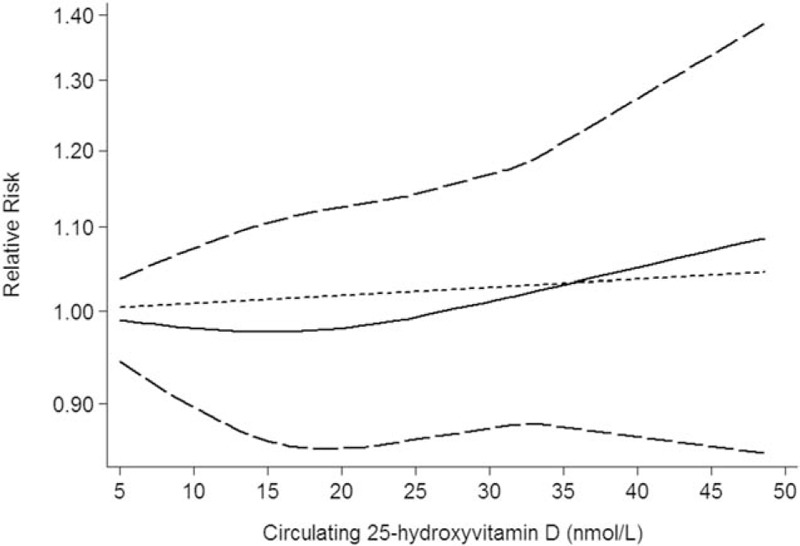
Circulating 25-hydroxyvitamin D is associated with lung cancer survival.

Five studies including 5 independent reports investigated the association between circulating 25-hydroxyvitamin D and lung cancer survival. Higher circulating 25-hydroxyvitamin D did not significantly decreased risk of lung cancer survival (RR: 1.01; 95% CI: 0.88–1.16; *P* < .001; Table 3). We also obtained the best fit at an inflection point of 10 nmol/L in piecewise regression analysis, increasing 10 nmol/L of circulating 25-hydroxyvitamin D was not increase lung cancer survival, the summary relative risk of lung cancer survival for an per 10 nmol/L of circulating 25-hydroxyvitamin D was 1.04 (95% CI: 0.91–1.17, *P* = .827; Fig. 4).

### 3.6 Subgroup analyses

Subgroup analysis was performed to check the stability of the primary outcome. Subgroup meta-analyses in study quality, number of participants, and number of cases showed consistent findings (Table 3).

### 3.7 Publication bias

The results show that no obvious evidence of publication bias was found in the associations between circulating 25-hydroxyvitamin D and lung cancer risk (Supplementary Table 1). *P* > .05 was considered no publication bias. A funnel plot for publication bias assessment is shown in Supplementary Figures 1 to 3.

## 4 Discussion

Vitamin D is an important vitamin, mainly from fat-rich fish, butter, cheese, and fortified milk. The body itself can produce vitamin D in the sun. However, vitamin D deficiency is a common phenomenon.^[8]^ It can maintain the stability of serum calcium and phosphorus levels, when the serum calcium concentration is low, it induced parathyroid hormone secretion, release it to the kidney and bone cells.^[33]^ Also, vitamin D participates in critical cell functions such as cell proliferation, apoptosis, differentiation, metastasis, and angiogenesis. Vitamin D is one of the indispensable elements of health and disease prevention. Previous studies supported higher circulating 25-hydroxyvitamin D significantly decrease lung cancer risk and survival. However, the result remains controversial.

In the current, meta-analysis was based on 17 prospective cohort studies, with 138,858 participants with 4368 incident cases. Thus, this meta-analysis provides the most up-to-date epidemiological evidence supporting higher circulating 25-hydroxyvitamin D is helpful for lung cancer. A dose–response analysis revealed that increasing 10 nmol/L dose of circulating 25-hydroxyvitamin D was associated with an 8% reduction in the risk of lung cancer risk and a 7% reduction in the risk of lung cancer mortality. Subgroup meta-analyses in study quality, number of participants, and number of cases showed consistent with the primary findings.

Several plausible pathways may reasonable for the relationship between 25-hydroxyvitamin D and lung cancer. Vitamin D metabolites play a cytostatic effect most dependent on vitamin D receptor. Previous study found that 25-hydroxyvitamin D plays the role of inhibiting lung cancer cells growth in mouse epidermal cells formation.^[34]^ The immunomodulatory function of vitamin D metabolites may be an important mechanism for vitamin D in prevent lung cancer; 25-hydroxyvitamin D can inhibit the activity of mammalian target of rapamycin in lung cancer cells and raise the level of protein expression, which can promote the autophagy of tumor cells.^[35]^ Meanwhile, 25-hydroxyvitamin can induce the expression of major antioxidant protein–superoxide dismutase SOD1 and SOD2, thereby inhibiting the formation of lung cancer to some extent.^[36]^ In addition, vitamin D can regulate immunological function of lung epithelial cells and inhibit cellular proliferation and angiogenesis while promoting cellular differentiation and apoptosis.^[34,37,38]^

Some limitations must be considered in this meta-analysis. First, we only select literature written in English, which may have resulted in a language or cultural bias, other languages should be chosen in the further. Second, we only select literature from PubMed and Embase databases, other databases should be chosen in the further.

In conclusion, our findings underscore the notion that higher vitamin D was significantly associated with lung cancer decrement. In the future, large-scale and population-based association studies must be performed in the future to validate the risk identified in the current meta-analysis.

## Supplementary Material

Supplemental Digital Content
