# Supplementary material for: Circulating 25-hydroxyvitamin D and lung cancer risk and survival: A dose–response meta-analysis of prospective cohort studies
Source: Medicine (Baltimore). 2017 Nov 10;96(45):e8613. doi: 10.1097/MD.0000000000008613 (PMC5690785; doi:10.1097/MD.0000000000008613)

**Supplementary table 1**. Publication bias analysis of the meta-analysis

|  | Test | t | 95% CI | P |
| --- | --- | --- | --- | --- |
| Circulating vitamin D and lung cancer risk | Begg’s test |  |  | 0.238 |
|  | Egger’s test | 0.64 | -0.26,1.05 | 0.329 |
| Circulating vitamin D and lung cancer mortality | Begg’s test |  |  | 0.931 |
|  | Egger’s test | 1.28 | -2.97,2.87 | 1.000 |
| Circulating vitamin D and lung cancer survival | Begg’s test |  |  | 0.113 |
|  | Egger’s test | 0.90 | -1.25,2.79 | 0.541 |

**Supplementary figure 1: A funnel plot for the meta-analysis of circulating 25-hydroxyvitamin D and lung cancer risk**


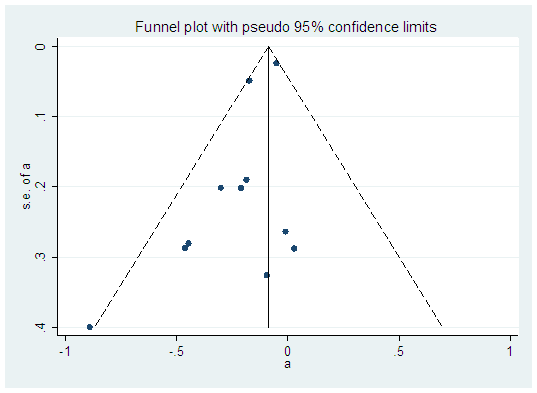


**Supplementary figure 2: A funnel plot for the meta-analysis of circulating 25-hydroxyvitamin D and lung cancer mortality**


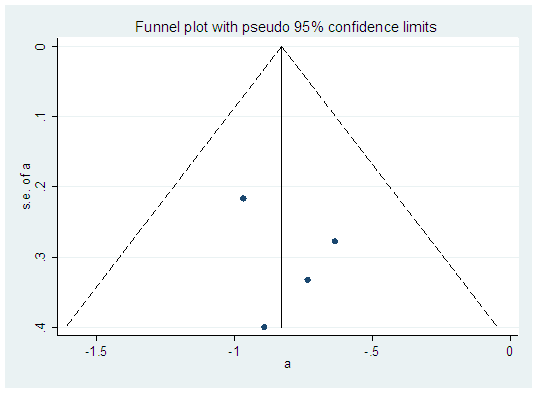


**Supplementary figure 3: A funnel plot for the meta-analysis of circulating 25-hydroxyvitamin D and lung cancer survival**


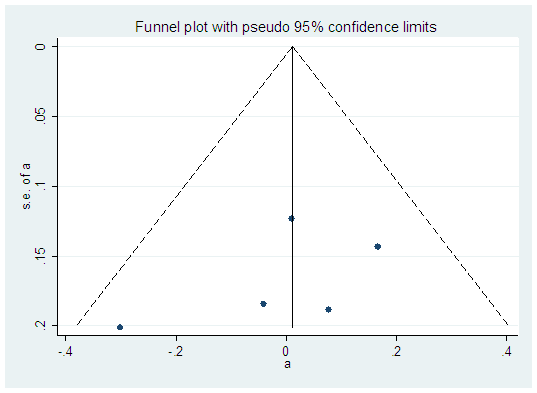

Supplement: Supplemental Digital Content [file medi-96-e8613-s001.doc]
